# The relationship between marital reminiscence styles and psychological well-being through mediating role of marital quality

**DOI:** 10.3389/fpsyg.2025.1639240

**Published:** 2025-08-26

**Authors:** Mohammad Reza Majzoobi, Simon Forstmeier

**Affiliations:** Developmental Psychology and Clinical Psychology of the Lifespan, University of Siegen, Siegen, Germany

**Keywords:** marital reminiscence styles, marital quality, psychological well-being, married people, Iran

## Abstract

**Introduction:**

Marital relationships are deeply shaped by the memories couples share, as reminiscence plays a pivotal role in fostering emotional connection and intimacy. Investigating how such reminiscence is related to marital quality provides valuable insights into its influence on relational dynamics. Therefore, the purpose of the present study was to examine the relationship between marital reminiscence styles (MRSs) and psychological well-being (PWB) through the mediating role of marital quality.

**Methods:**

This was a descriptive-correlational study. The statistical population included all married people living in Kermanshah, Iran in 2023, among whom a sample of 304 people were selected using convenience sampling method. The measures used in this study were Majzoobi and Forstmeier’s (2025) marital reminiscence styles Questionnaire (MRSQ), Chonody et al.’s (2018) Relationship quality Questionnaire, and Ryff’s PWB Questionnaire. Data were analyzed through Pearson’s correlation coefficient and structural equations modeling (SEM) in SPSS-26 and LISREL-10 software.

**Results:**

The findings indicated that the hypothesized model had a good fit in the studied sample. MRSs were significantly associated with PWB through marital quality. As such, it was found that obsessive MRS is related negatively to marital quality, which, in turn, related positively to PWB. Moreover, narrative MRS was related positively to marital quality, which, in turn, related positively to PWB.

**Discussion:**

These results underscore the importance of fostering positive MRSs, such as narrative MRS, to enhance marital quality and PWB in marital relationships.

## Introduction

Psychological well-being (PWB) is a critical construct for assessing individuals’ adaptive functioning in life. PWB is defined as positive intrapersonal and interpersonal functioning, characterized by relatedness with others, self-referent attitudes that foster a sense of personal growth and mastery (Burns, 2016). As this definition suggests, PWB is shaped by both individual and relational factors. Research has demonstrated that intrapersonal variables, such as personality traits (Wilson et al., 2024) and self-esteem (Zell and Johansson, 2024), as well as interpersonal variables, including empathy (Swenson et al., 2024) and social interaction skills (Lamash et al., 2024), are significant predictors of PWB. Among the interpersonal factors, marital interaction stands out as particularly influential. Studies have shown that marital intimacy (Aghamiri and Vaziri, 2019) and marital quality (Carr et al., 2014) play pivotal roles in predicting individuals’ PWB, highlighting the centrality of marital dynamics in overall mental health.

Marital quality is a key construct in evaluating the state of marital relationships. It is defined as spouses’ subjective feelings, which manifest themselves in the form of evaluative judgments about their marriage and partner (Fincham and Bradbury, 1987). One critical determinant of marital quality appears to be the level of interaction between spouses. To the extent that Johnson et al. (1986), in his two-factor model, posits that marital quality consists of positive factors (such as marital satisfaction and interaction) and negative factors (such as disagreements, marital problems, and instability). According to Johnson, the extent of marital interaction, reflected in the frequency and depth of communication between spouses, is a pivotal determinant of marital quality. Similarly, Allendorf and Ghimire (2013) identified five factors in their model of marital quality, including satisfaction, communication, togetherness, problems, and disagreements. Among these, communication, which encompasses the frequency of interactions and discussions on various topics, plays a central role. Thus, the degree of interaction and dialogue between spouses may consider as significant factors in their marital relationship. A fundamental database for these interactions is the shared memories that couples have built throughout their marital life, commonly referred to as marital reminiscence (MR).

MBR refers to memories associated with the marital relationship, formed during the course of the couple’s shared life, which are vividly remembered and often carry strong emotional significance. Studies have shown that MBR is correlated to positive marital outcomes such as marital satisfaction (Alea and Vick, 2010; Amani et al., 2024; Bazzini et al., 2007; Doohan et al., 2010; Dunlop, 2019; Lenger and Gordon, 2019; Mallory et al., 2018; McCoy et al., 2017), marital well-being (Holmberg et al., 2018), and marital quality (Philippe et al., 2013). In a meta-analysis, Majzoobi and Forstmeier (2022) reported substantial effect sizes for the relationship between MBR and positive marital outcomes in general (*r* = 0.334) as well as marital satisfaction in particular (*r* = 0.445). These findings highlight that MBR is positively associated with various beneficial outcomes for couples.

Reminiscence can take different styles. Based on Watt and Wong’s (1991) model, which categorized reminiscence in older people into six styles, namely integrative, narrative, problem-solving, transmissive, obsessive, and escapist, Majzoobi and Forstmeier (2025) adapted the framework to develop a model of marital reminiscence styles (MRSs) for married individuals. Their factor analysis yielded a four-factor model encompassing reminiscence frequency, obsessive reminiscence style (OBRS), narrative reminiscence style (NRS), and integrative-problem-solving reminiscence style (IPSRS). This model provides a novel perspective on how couples engage with and utilize MR in their relational dynamics. In this model, the reminiscence frequency implies the extent of using and deriving pleasure from MR. OBRS refers to the continuous and unconstructive review of negative MR. NRS indicates the extent to which couples use MR for interaction, communication with each other, and creating intimacy. IPSRS involves using past memories to reach meaning and value in their relationship as an integrate marital bond, and to find solutions to current relationship issues.

### Age and gender differences in MR

A key aspect of MR involves how individual factors, such as gender and age, shape MRSs and their effects on relationships. Findings on gender’s role in MR are mixed. On the one hand, some studies (e.g., Alea and Bluck, 2007; Lenger and Gordon, 2019; Philippe et al., 2013) found no significant moderating effect, suggesting men and women benefit similarly from recalling Relationship-defining memories (RDMs). Other research shows women are more sensitive to RDMs, with stronger effects on marital intimacy (e.g., Alea and Vick, 2010; Guilbault and Philippe, 2017). Women more often recall detailed, emotional, and rehearsed memories. Their storytelling skills have been linked to both their own and their husbands’ marital satisfaction (McCoy et al., 2017). On the other hand, some studies highlight men’s significant role. Men who recall memories in a structured, emotionally engaged way report less distress and more marital satisfaction (e.g., Holmberg et al., 2018; Koenig Kellas et al., 2010; McCoy et al., 2017). This may relate to their problem-focused coping and the benefits of structured memory narration, such as writing (Graybeal et al., 2002; Smyth, 1998).

Regarding age, some studies (e.g., Alea and Bluck, 2007; Lenger and Gordon, 2019) found no moderating effects. However, others report higher RDMs rehearsal in the 20–29 and 70–85 age groups, and the strongest link between emotional intensity and marital satisfaction among those aged 50–69 (Alea and Vick, 2010). Philippe et al. (2013) also found that in younger individuals, need satisfaction in memories is more strongly tied to marital quality, suggesting this influence weakens with age.

### The current study

**Figure 1:**
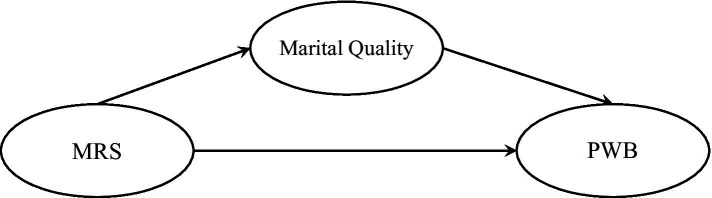
The hypothesized model of the relationship between MRSs and PWB through marital quality.

As outlined in previous sections, research over the past years has extensively examined the relationships between MBR and marital quality, as well as between marital quality and PWB. These pairwise associations have been consistently supported by empirical findings. However, it is now crucial to move beyond these dyadic relationships and develop a structural model that integrates MBR, marital quality, and PWB. To the best of our knowledge, no prior study has explored these three variables within a single framework. Nevertheless, Amani et al. (2024) demonstrated that storytelling in marital relationships and narrative mindset (a construct closely aligned with MR) mediate the relationship between attachment styles and marital satisfaction among married individuals. A notable gap in this area of research has been the absence of a robust tool to assess the frequency and styles of MBR. With the recent development of such a measure, it is now possible to test comprehensive models involving this construct. Addressing this research gap is of significant importance, not only to advance theoretical understanding but also to guide practitioners in designing marital therapy protocols that emphasize MBR interventions. Such investigations could shed new light on the nuances of MR and open chapters for further exploration. In light of this research gap and the potential practical implications, the present study aimed to examine the relationship between MRSs and PWB through the mediating role of marital quality. The hypotheses of the current study were (1) MRSs are associated with marital quality, (2) MRSs are associated with PWB, (3) MRSs are related to PWB through the mediating role of marital quality, (4) There is a difference in MRSs across gender groups, and (5) There is a difference in MRSs across age groups (see Figure 1).

## Method

### Study design and participant

This was a descriptive-correlational study using structural equations modeling (SEM) to explore the research objectives. The population included all married individuals in Kermanshah, Iran, among whom 304 participants were selected using convenience sampling. Regarding sample size, Stevens (1996), as cited in Momeni et al. (2021) recommended a rule of thumb of 15 cases per predictor variable in multiple regression analysis, which is considered robust for ordinary least squares methods. Given the similarities between SEM and multivariate regression, particularly in some methodological aspects, using a minimum of 15 cases per measured variable in SEM is deemed reasonable (Hooman, 2014). Additionally, Loehlin (1992), as cited in Momeni et al. (2022) suggested that for models with two to four factors, researchers should aim to collect at least 100 cases, with a preference for 200 or more. Based on these recommendations, the sample size in this study was sufficient for SEM analysis.

Inclusion criteria for the study were as follows: (1) being married, (2) age between 20 and 70 years, (3) having been in the current marital relationship for at least 1 year, and (4) providing informed consent to participate. Exclusion criteria included (1) experiencing the loss of a loved one in the past 3 months and (2) men having two or more concurrent spouses. Participants ranged in age from 21 to 70 years (M = 45.58, SD = 14.01), with the duration of their marriages ranging from 1 to 50 years (M = 18.74, SD = 12.54). Among the participants, 39 individuals (12.8%) had no children, 33 (10.9%) had one child, 69 (22.7%) had two children, 67 (22%) had three children, and 96 (31.6%) had more than three children. Regarding educational attainment, one participant (0.3%) was illiterate, 25 (8.2%) had less than a high school diploma, 70 (23%) held a high school diploma, 65 (21.4%) had an associate degree, 137 (45.1%) held a bachelor’s degree, and six (2%) had a master’s degree.

### Measures

#### The Marital Reminiscence Questionnaire (MRQ)

MRQ was developed by Majzoobi and Forstmeier (2025) to assess the frequency and styles of reminiscence within marital relationships. Grounded in Watt and Wong’s (1991) categorization of reminiscence, the MRQ comprises 21 items distributed across four factors of reminiscence frequency (3 items), OBRS (6 items, negative), NRS (4 items, positive), and IPSRS (8 items, positive). Each item is rated on a 5-point Likert scale, ranging from 1 (Never) to 5 (Always). Total scores of the MRQ subscales range from 21 to 105, with higher scores on each subscale indicating greater prevalence of the corresponding style in marital relationships. Majzoobi and Forstmeier reported satisfactory internal consistency for the subscales, with Cronbach’s alpha coefficients of 0.72, 0.80, 0.71, and 0.80 for reminiscence frequency, OBRS, NRS, and IPSRS, respectively. The questionnaire’s validity was examined through confirmatory factor analysis (CFA), convergent validity, and predictive validity. CFA results supported the four-factor structure, demonstrating good model fit in the study sample. Convergent validity was indicated by significant positive correlations between the two positive subscales (NRS and IPSRS) and positive reminiscence functions from Webster’s (1997) model, alongside significant negative correlations with negative reminiscence functions. The OBRS subscale showed a significant positive correlation with the subscale of bitterness revival. Test–retest reliability over a one-month interval revealed high temporal stability, with coefficients ranging from 0.83 to 0.89 for the four subscales. These findings suggest that the MRQ is a reliable and valid tool for assessing MRSs. CFA of the MRQ was conducted on the same sample used in the present study, as part of a larger research project. The key results of this analysis are briefly reported above to support the factorial validity of the instrument. A more detailed presentation of the CFA findings is provided in a separate article currently under publication.

#### Relationship Quality Scale (RQS)

Chonody et al. (2018) designed this tool and conducted a study on 8,132 samples from the United Kingdom, the United States, and Australia to determine its validity and reliability. Initially, the tool had 15 items, and after EFA, the final scale consisted of 9 items and one factor. Each item has a 5-point Likert scale ranging from 1 = completely impossible to 5 = completely agree. The maximum and minimum scores in this questionnaire are 45 and zero, respectively, with higher scores indicating higher relationship quality. The reliability of this tool, assessed by Cronbach’s alpha coefficient, was 0.90, indicating high internal consistency (Chonody et al., 2018). In Iran, Taqizadeh Firouzjaei et al. (2018) conducted a CFA for the Farsi version of this questionnaire, showing that the single-factor structure of this questionnaire has appropriate validity. They reported the reliability of this questionnaire as 0.90 based on the Cronbach’s alpha coefficient. In the present study, the reliability of this questionnaire, based on the Cronbach’s alpha coefficient, was 0.75 in the sample one and 0.65 in the sample two.

#### Psychological Well-Being Questionnaire (PWBQ)

PWBQ was developed by Ryff (1989) and consists of 18 items. The questionnaire measures six subscales of purpose in life, positive relations with others, personal growth, self-acceptance, autonomy, and environmental mastery. Responses are recorded on a 6-point Likert scale ranging from 1 (strongly disagree) to 6 (strongly agree). Total scores range from 18 to 108, with higher scores indicating greater levels of PWB. Ryff and Singer (2006) reported correlations ranging from 0.79 to 0.89 between the 18-item version and the original 84-item scale. In Iran, Sefidi and Farzad (2012) standardized the 18-item version and reported internal consistency reliability coefficients for the total scale and subscales ranging from 0.65 to 0.75. Additionally, Khanjani et al. (2014) demonstrated strong model fit for the six-factor structure through confirmatory factor analysis (CFA) in a sample comprising both genders. Similarly, Van Dierendonck (2004) found satisfactory internal consistency for the subscales, with Cronbach’s alpha coefficients ranging from 0.77 to 0.90. In the present study, the Cronbach’s alpha coefficients for the questionnaire ranged between 0.78 and 0.83, indicating good reliability.

### Procedure

Following approval from the Ethics Committee of Kermanshah University of Medical Sciences, Iran (IR. KUMS. REC.1402.066), the preliminary phase of the study was conducted. During this phase, the study questionnaires were designed as online surveys hosted on the Porsall platform, a knowledge-based company in Iran specializing in data collection for scientific research. The process involved uploading the questionnaire package, along with response options, onto the platform, which then generated a unique link for the survey. Participants could access the survey by clicking on the provided link, redirecting them to the questionnaire page for completion. Once the questionnaires were finalized, the company distributed invitations via social media networks across Kermanshah Province, Iran, to recruit participants. Before proceeding to the survey, participants were informed about the study’s objectives, assured of confidentiality and anonymity, and asked to provide informed consent. Eligibility criteria were assessed through preliminary screening questions, and only those meeting the inclusion criteria were granted access to the full questionnaire. Ultimately, 304 participants from the target population completed the surveys. The collected data were analyzed using Pearson’s correlation coefficient, two-way ANOVA and Welch’s ANOVA in SPSS-21 and SEM in LISREL-10.

## Results

To examine the effects of gender and age on different styles of marital reminiscence, a series of two-way ANOVA and Welch’s ANOVA tests were conducted. The results showed that neither gender nor age had a significant effect on reminiscence frequency, and no interaction effect was observed. Scores were nearly identical for males (*M* = 6.58, SD = 1.92) and females (*M* = 6.64, SD = 1.80), and no significant differences were found between age groups (all *p* > 0.05). Similarly, for obsessive reminiscence, no significant main or interaction effects were found. Gender (*F*(1, 294) = 0.117, *p* = 0.733, η^2^ = 0.000), age group (*F*(4, 294) = 0.447, *p* = 0.774, η^2^ = 0.006), and the interaction (*F*(4, 294) = 0.552, *p* = 0.697, η^2^ = 0.007) were all non-significant. Mean scores were similar between males (*M* = 13.83, SD = 3.13) and females (*M* = 13.95, SD = 2.91).

In contrast, NRS was significantly affected by age (*F*(4, 294) = 7.298, *p* < 0.001, η^2^ = 0.090), with younger participants reporting higher scores. The highest mean was observed in the 21–30 age group (*M* = 10.38, SD = 2.12), and the lowest in the 61–70 age group (*M* = 8.73, SD = 2.13). The difference between these two groups was statistically significant (*p* < 0.001), while comparisons between the 31–40 and 51–60 age groups (*p* = 1.000) and 51–60 and 61–70 age groups (*p* = 1.000) were not, indicating a stabilization of decline in later adulthood. Gender had no significant effect (*F*(1, 294) = 0.852, *p* = 0.357, η^2^ = 0.003), nor did it moderate the effect of age (*F*(4, 294) = 0.318, *p* = 0.866, η^2^ = 0.004).

**Figure 2:**
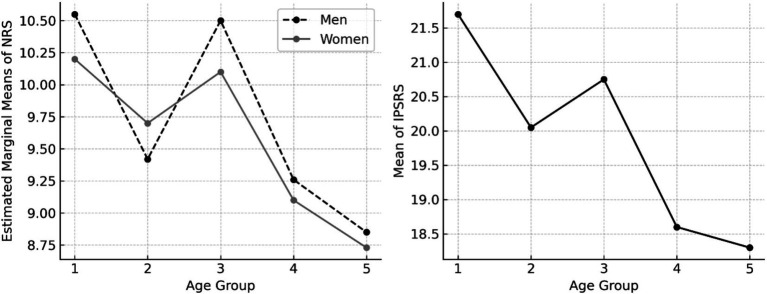
Age-related differences in NRS and IPSRS across gender. IPSRS, Integrative-Problem Solving Reminiscence; NRS, Narrative reminiscence Style.

For IPSRS, Welch’s ANOVA (due to a violation of the homogeneity of variance assumption) revealed a significant main effect of age (*F*(4, 148.783) = 7.564, *p* < 0.001). Post-hoc Games-Howell tests showed that the 21–30 age group (*M* = 21.65, SD = 4.37) scored significantly higher than the 51–60 (*M* = 18.64, SD = 3.81, *p* = 0.023) and 61–70 (*M* = 18.27, SD = 3.81, *p* = 0.004) age groups, and the 41–50 age group (*M* = 20.78, SD = 4.34) also scored significantly higher than the 51–60 (*p* = 0.023) and 61–70 (*p* = 0.004) age groups. No significant differences were found between the 51–60 and 61–70 age groups (*p* = 0.982), indicating a plateau in older adulthood. Gender had no significant effect on IPSRS (*F*(1, 301.777) = 1.217, *p* = 0.271), although men (*M* = 20.20) scored slightly higher than women (*M* = 19.80). The results can be seen in Figure 2.

Prior to the analysis, SEM assumptions including distribution normality, error independence, and multiple alignment were examined. To examine the normality of the research variables, the skewness and kurtosis of the distribution of scores were used, the results of which showed that the distribution of scores of all variables is normal (with the range of distribution between +1.5 and −1.5). The Dorbin-Watson test was used to evaluate the independence of the errors, which showed no correlation between the errors (D. W = 1.64, range between 1.5 to 2.5 is acceptable) variance Inflation (VIF) and tolerance were used to evaluate the multiple alignment between the predictor variables. The results showed that there is no alignment between the variables (VIF amplitude less than 5 and tolerance higher than 0.1). The results of these assumptions are presented in Table 1 along with the mean and standard deviation of the variables.

**Table 1:** The mean, standard deviation, and information regarding assumptions of SEM.

Variable	Min	Max	*M*	SD	Skewness	Kurtosis
Marital reminiscence styles						
The extent of reminiscence	3	13	6.41	2.03	0.394	−0.193
Obsessive reminiscence	8	30	23.31	4.98	−0.774	0.096
Narrative reminiscence	4	16	9.61	2.81	0.082	−0.725
Integrative-problem solving reminiscence	8	34	20.42	4.67	−0.115	−0.276
Marital quality	9	45	15.56	4.84	1.526	1.096
Psychological well-being	34	83	57.81	7.45	−0.927	0.959
Autonomy	5	15	9.54	1.53	0.063	0.52
Environmental mastery	3	16	9.22	2.13	−0.428	0.346
Personal growth	3	17	9.05	2.06	−0.4	0.818
Positive relations with others	4	15	10.64	2.17	−0.544	0.156
Purpose in life	4	16	10.72	1.89	−0.562	0.378
Self-acceptance	3	18	8.64	2.07	−0.082	1.003

*n* = 304.

Another assumption is the establishment of a linear relationship between independent and dependent variables, which was examined by Pearson’s correlation coefficient, the results of which are reported in Table 2.

**Table 2:** The correlation coefficient between the variables.

Variable	1	2	3	4	5
1. RF	–				
2. OBRS	−0.13	–			
3. NRS	0.349^**^	−0.039	–		
4. IPSRS	0.419^**^	0.149^**^	0.627^**^	–	
5. MQ	0.11	−0.146^*^	0.253^**^	0.214^**^	–
6. PWB	0.157^**^	−0.245^**^	0.170^**^	0.142^*^	0.372^**^

^*^p < 0.05, ^**^p < 0.01.

Au, Autonomy; EM, Environmental mastery; IPSRS, Integrative-Problem Solving Reminiscence; MQ, Marital Quality; NRS, Narrative reminiscence; OBRS, Obsessive Reminiscence; PG, Personal growth; PIL, Purpose in Life; PRWO, Positive Relations with Others; PWB, Psychological Well-Being; RF, Reminiscence Frequency; SA, Self-acceptance.

**Figure 3:**
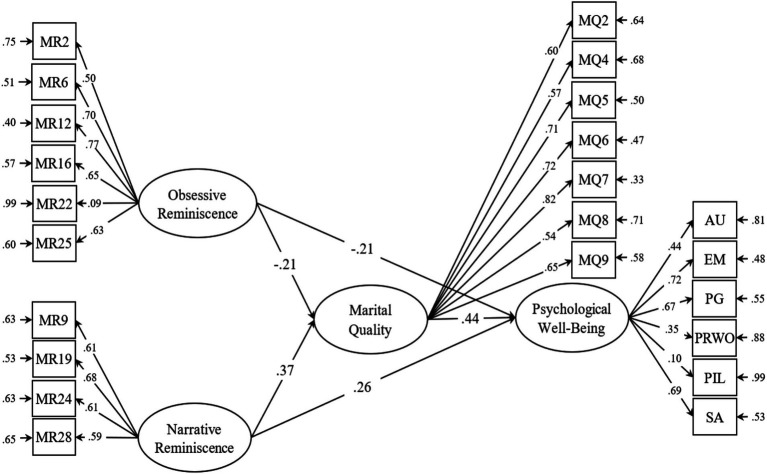
The final model of the relationship between MRSs and PWB through marital quality.

Table 2 shows the correlation between the research variables in that most of the relationships between the variables are significant at the significance level of 0.01. Correlation analysis provides insight into the two-way relationships between research variables. In order to simultaneously test the relationship between variables in the present study, SEM was used, the output model of which can be seen in Figure 3. Table 3 shows the fitness indices of the output model of the study along with expected indices. According to Table 3, used to determine the adequacy of the proposed model with the data were a combination of fitness indicators such as chi-square (*χ*^2^), normalized chi-square measure (chi-square ratio of degrees of freedom), good fit indices (GFI), normalized fit (NFI), adaptive fit (CFI) and root mean squared error approximation (RMSEA).

**Table 3:** Fit indices for the developed model.

Model fit indices	*X* ^2^	df	*X*^2^/df	GFI	AGFI	NFI	IFI	CFI	RMSEA
Obtained values	360.41	219	1.64	0.92	0.90	0.91	0.96	0.96	0.046

According to the information in Table 3, the degree of fitness indicators such as incremental fitness index (IFI = 0.96), compatibility fitness index (CFI = 0.96), normalized fitness index (NFI = 0.91) as well as index of the root mean squared error approximation (RMSEA = 0.046) indicates a very good fit of the model with the data. Other fit indices such as good fit index (GFI = 0.92) and adjusted good fit index (AGFI = 0.90) are also acceptable. Table 4 shows the parameters related to the direct effects of variables on each other in the proposed research model.

**Table 4:** Coefficients of the model of the relationship between MRSs and well-being through marital quality.

Direct path	*β*	Statistic- *t*
OBRS → MQ	−0.16	−3.04^**^
NRS → MQ	0.39	4.51^**^
OBRS → PWB	−0.62	−3.14^**^
NRS → PWB	1.17	3.26^**^
MQ → PWB	1.18	5.38^**^

^**^p < 0.01.

NRS, Narrative reminiscence Style; MQ, Marital Quality; OBRS, Obsessive Reminiscence Style; PWB, Psychological Well-Being.

As the results in Table 4 show, the direct paths are significant. The Sobel test was also used to investigate the mediating role of marital quality in the relationship between MRSs and PWB, the results of which are reported in Table 5.

**Table 5:** Sobel test for the mediating role of marital quality in the relationship between MRSs and PWB.

Variables	*p*	Sobel’s test (z)
OBRS → MQ → PWB	*p* < 0.001	3.17^**^
NRS → MQ → PWB	*p* < 0.001	3.80^**^

NRS, Narrative reminiscence Style; MQ, Marital Quality; OBRS, Obsessive Reminiscence Style; PWB, Psychological Well-Being.

According to Table 3, the proposed research model has desirable fitness indicators. The root mean squared error approximation (RMSEA = 0.046) indicates a very good fit of the model with the data. Therefore, the proposed model has a good fit in the sample. According to the information in Table 5, the significance of the Sobel test indicates the significance of the mediating role of marital quality in the relationship between MRSs and PWB.

## Discussion

Marital relationships play a crucial role in individuals’ PWB, serving as a foundation for emotional support and life satisfaction. One significant but often overlooked aspect of marital interactions is the role of MR, where couples reflect on shared memories to strengthen their bond. Such MRSs can influence relational dynamics and, in turn, individual PWB. Therefore, the present study aimed to investigate the relationship between MRSs and PWB through the mediating role of marital quality. The findings demonstrated that the hypothesized model exhibited good fit within the studied sample. In this model, both OBRS and NRS were significantly associated with PWB through marital quality.

The study proposed three hypotheses. The first hypothesis, stating that MRSs are associated with marital quality, was partially confirmed. Specifically, individuals with an OBRS tend to experience lower marital quality, while those with a NRS report higher marital quality. This finding aligns with previous studies (Alea and Vick, 2010; Amani et al., 2024; Bazzini et al., 2007; Doohan et al., 2010; Dunlop, 2019; Lenger and Gordon, 2019; Mallory et al., 2018; Philippe et al., 2013). The second hypothesis, proposing that MRSs are associated with PWB, was also partially confirmed. Individuals with an OBRS tend to experience lower PWB, whereas those with a NRS report higher PWB. This result is consistent with prior research, such as Holmberg et al. (2018). The third hypothesis, which suggested that MRSs are associated with PWB through marital quality, was partially confirmed. Specifically, individuals with an OBRS experience lower marital quality, which in turn is significantly associated with reduced PWB. Notably, this hypothesis had not been examined in prior studies. Overall, these findings suggest that individuals who obsessively ruminate on negative and distressing marital memories tend to experience lower marital quality, which subsequently contributes to diminished PWB. In contrast, those who engage in a NRS about marital memories, using them as a means to foster intimacy and connection with their spouse, report higher marital quality, which is associated with greater PWB. Besides, the fourth hypothesis, which proposed a difference in MRSs based on gender, was not confirmed and gender had no significant effect on any of the reminiscence styles. However, the fifth hypothesis, regarding age-related differences, was partially confirmed. As such, there were significant differences in NRS and IPSRS across age groups, but no significant differences in frequency or OBRS.

### The relationship between MRSs and marital quality

The models discussed in this context do not directly address obsessive reminiscence of negative marital memories or narrative reminiscence, but based on these models, we believe that both types of reminiscence can have a powerful influence on marital quality in different ways. For instance, according to the social bond model (Bazzini et al., 2007), when couples obsessively recall negative memories, it might disrupt the emotional disclosure process that’s so crucial for maintaining intimacy. Imagine a couple who frequently revisits arguments or past disappointments, they may begin to feel emotionally distant rather than close. This constant rumination on past hurts could create a barrier to emotional connection, making it harder for them to rebuild trust or bond. In contrast, NRS, where couples use their shared marital memories to interact and communicate, helps strengthen their emotional bond. For instance, when partners talk about fond moments from their past, like their first trip together or the early days of their relationship, they feel more connected. This positive communication enhances intimacy and trust, which is vital for marital quality.

Following the self-expansion model [Aron and Aron, 1997, as cited in Bazzini et al. (2007)], obsessive reminiscence of negative memories might prevent couples from appreciating their shared growth. Picture a couple who fixates on past struggles or conflicts—they may start to feel stuck, unable to see how much they have grown together over time. This focus on the negative can inhibit the emotional connection and prevent the couple from experiencing the shared understanding that helps them thrive. On the other hand, NRS gives couples the opportunity to reflect on the good times they have shared, reinforcing their sense of togetherness. By reminiscing about positive experiences, like moments of mutual support or joy, couples deepen their connection and emotional bond, which enhances marital satisfaction.

From the perspective of self-determination theory (Deci and Ryan, 2000), obsessive rumination on negative memories could hinder the satisfaction of the basic psychological needs for autonomy, competence, and relatedness. For instance, if a partner constantly rehashes past mistakes, they may begin to feel like their emotional needs are not being met, leading to frustration and dissatisfaction. When these needs go unmet, the relationship can start to feel draining and unfulfilling. In contrast, NRS offers couples the chance to meet these psychological needs. Through sharing positive memories, such as moments of affection or achievements, partners experience a sense of relatedness and competence, which helps foster a stronger and more fulfilling connection, ultimately enhancing marital satisfaction.

In terms of the sense-making model (Koenig Kellas et al., 2010), OBRS may prevent couples from reframing past negative experiences in a way that could lead to growth. Think of a couple who keeps focusing on a painful argument or betrayal, unable to move past it. This rumination can prevent them from finding meaning in their challenges, keeping them stuck in unresolved emotional conflict. In contrast, NRS offers couples the opportunity to reinterpret past difficulties through a more positive lens. For example, discussing how they overcame challenges together or what they learned from past struggles can create a sense of mutual growth and understanding. This re-framing allows for better communication, healthier conflict resolution, and improved relational well-being.

Finally, from the self-regulation model (Frattaroli, 2006), obsessive revisiting of negative memories may interfere with emotional processing, making it harder for couples to manage their emotions and conflicts. For example, when couples continually dwell on past mistakes, it may be hard for them to manage current disagreements effectively. Without the ability to regulate emotions, they may struggle to communicate and resolve issues. On the other hand, NRS allows couples to process past emotions by revisiting positive memories together. Imagine a couple remembering a joyful event like the birth of their child or a time when they worked through a difficult situation as a team. These shared recollections provide emotional stability and help partners regulate their emotions, reinforcing a sense of emotional connection and stability, which contributes to a healthier relationship.

In summary, based on these models, we believe that obsessive reminiscence of negative memories can have a detrimental effect on marital quality by preventing emotional connection, hindering growth, and reducing satisfaction. In contrast, NRS, by fostering positive communication, emotional regulation, and mutual understanding, can enhance marital quality by reinforcing emotional bonds, promoting relational growth, and satisfying psychological needs.

### The relationship between MRSs and well-being

When it comes to the relationship between reminiscence and PWB, the way couples reminisce about their past plays a significant role. For couples who engage in NRS, where they share and reflect on positive marital memories, these recollections help strengthen their emotional connection. They use these memories as a way to interact, communicate, and build intimacy, which can foster feelings of closeness and understanding. According to the social bond model (e.g., Bazzini et al., 2007), this process promotes social bonds that increase marital intimacy and, by extension, life satisfaction. As couples reflect on happy moments together, they also engage in deeper communication, which enhances the sense of personal growth, autonomy, and mastery over their relationship dynamics. Additionally, the self-expansion model [Aron and Aron, 1997, as cited in Bazzini et al. (2007)] suggests that recalling these positive memories fosters a shared sense of growth and connection, reinforcing marital satisfaction and contributing to PWB. This type of reminiscence provides couples with opportunities to reevaluate their shared experiences, which deepens mutual understanding and creates a supportive environment that nurtures both individual and relational well-being.

On the other hand, when couples engage in OBRS, which focuses on negative memories and unresolved conflicts, the emotional impact can be quite different. This type of reminiscence can create emotional distance, as it revolves around past hurts and dissatisfaction. According to self-determination theory (Deci and Ryan, 2000), when psychological needs like autonomy and relatedness are unmet, it negatively affects life satisfaction and relational quality. OBRS can hinder emotional growth and obstruct the fulfillment of these needs, making couples feel less connected and less satisfied. Moreover, sense-making models (Koenig Kellas et al., 2010) suggest that when couples reflect on their difficult experiences, it can help them find meaning and resolution, but obsessive rumination tends to reinforce negative emotions and keeps them stuck in a cycle of distress. The constant replay of negative experiences not only diminishes life satisfaction but also undermines the couple’s ability to develop resilience, reducing overall PWB.

In essence, NRS can be a powerful tool for couples to reinforce their emotional bond and promote personal growth, contributing positively to their PWB, as suggested by the self-expansion and social bond models. However, OBRS, as outlined in self-determination theory and sense-making models, tends to hinder this growth and cause emotional distress, ultimately reducing both relationship quality and individual well-being. Couples who focus on positive memories tend to feel more connected and satisfied, while those stuck in obsessive rumination may experience a decline in their overall sense of meaning and life satisfaction.

### The relationship between MRSs and well-being through marital quality

The way couples reflect on their past experiences, whether they focus on negative memories or engage in positive reminiscence, has a significant impact on both the quality of their relationship and their PWB. For couples who tend to obsessively revisit negative memories, this rumination can create emotional distance and hinder relational growth. Consider a couple who frequently rehash unresolved conflicts, such as a betrayal or an argument that never fully healed. While they may intend to address the issue, their focus on the past prevents them from moving forward. The constant reliving of hurtful moments inhibits emotional connection and trust-building, making it harder for them to experience the closeness that is crucial for marital satisfaction. The more they dwell on these negative memories, the more disconnected they feel, and their relationship suffers as a result. This emotional disconnection may also leave both partners feeling that their psychological needs for autonomy, competence, and relatedness are unmet, ultimately leading to reduced PWB.

In contrast, couples who engage in NRS, where they recall and reflect on positive memories, experience a different dynamic. For example, imagine a couple recalling the joy they felt on their honeymoon or remembering the support they provided each other during a difficult time. Sharing such meaningful memories fosters positive communication, strengthens emotional bonds, and promotes intimacy. These moments of connection not only help partners feel closer to one another, but they also satisfy psychological needs such as a sense of relatedness and mutual growth. By reflecting on the positive experiences they have shared, couples can gain a deeper appreciation for their relationship, reinforcing their trust and emotional stability. This sense of mutual support and growth, in turn, enhances their overall well-being and marital satisfaction.

The difference between these two types of reminiscence is stark. When couples focus on negative memories, they risk getting stuck in the past, hindering their ability to build a future together. This obsessive rumination can undermine their emotional connection and prevent them from experiencing the growth and understanding necessary for a thriving relationship. On the other hand, NRS allows couples to not only relive positive moments but also to strengthen their bond, enhance their communication, and deepen their emotional connection, all of which contribute to greater PWB and marital quality.

**Figure 4:**
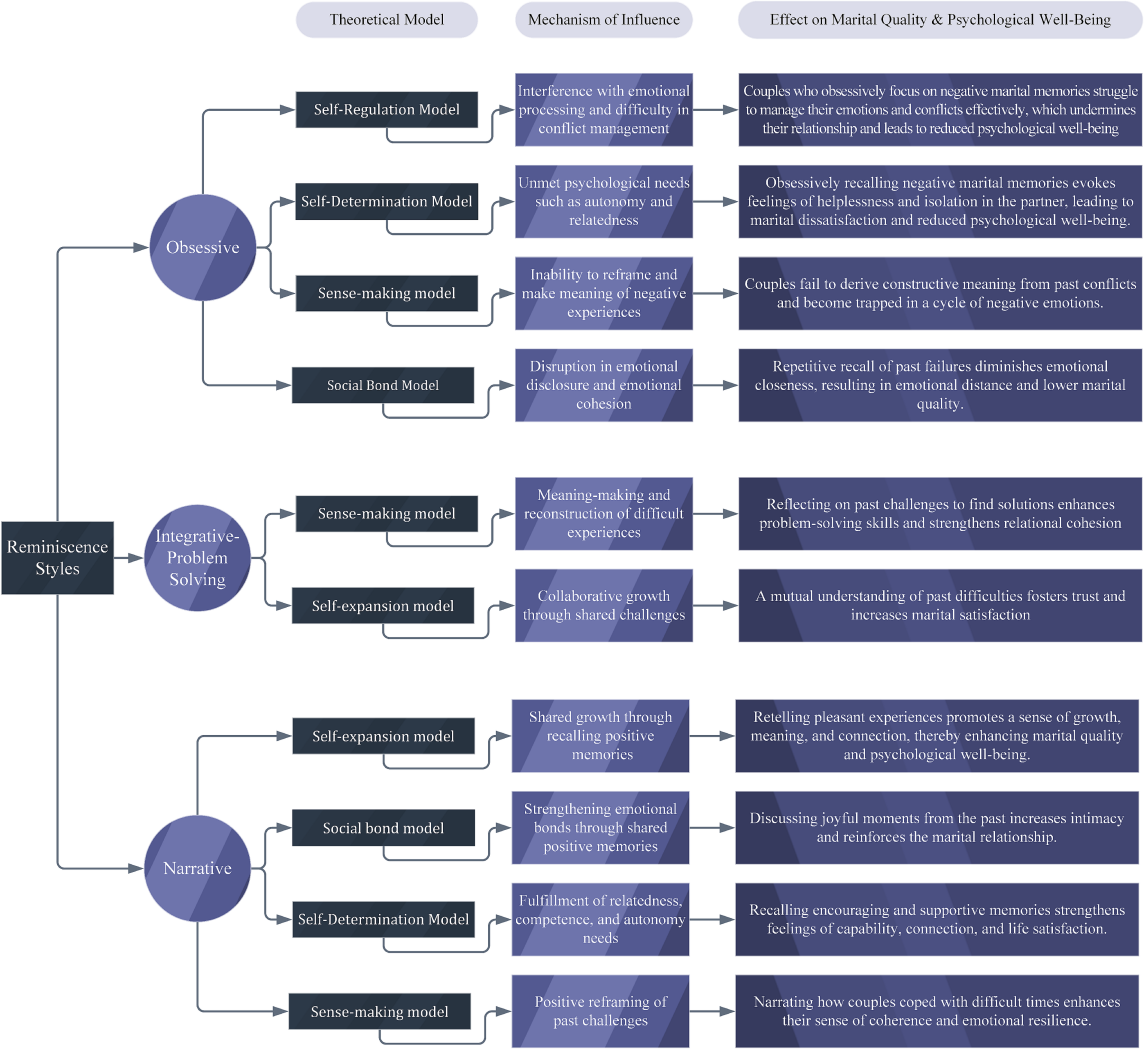
Theoretical framework explaining how MRSs may affect marital quality and PWB.

To summarize the explanatory interpretations discussed, a conceptual model has been developed and presented to illustrate how the aforementioned theoretical frameworks (namely the self-expansion model, sense-making model, self-regulation model, social bond model, and self-determination theory) may help explain the potential mechanisms through which different MRSs influence marital quality and psychological well-being (see Figure 4).

### The role of gender and age in MRSs

The findings of the study revealed that gender had no significant effect on any of the reminiscence styles, while age significantly influenced NRS and IPSRS, with younger adults reporting higher levels than older ones.

Although prior research has shown that gender may influence MR, with findings suggesting that women tend to produce more emotionally detailed and relational narratives (Alea and Vick, 2010; McCoy et al., 2017). The current study did not reveal significant gender differences in any of the MRSs which is in line with some previous studies (Lenger and Gordon, 2019; Philippe et al., 2013), suggesting that gender may not always play a moderating role in reminiscence-related processes. Several factors may explain this outcome. First, the use of structured self-report instruments in our study likely minimized gender variability. While open-ended narrative tasks often highlight stylistic and emotional differences between men and women, standardized Likert-type scales tend to constrain variation by guiding all respondents toward uniform answer formats (Grysman and Hudson, 2013). This may make men more likely to express reminiscence in a way similar to women. Second, the social dynamics of shared memory in marital life may attenuate gender effects, even when individuals are assessed separately. According to transactive memory theory (Wegner et al., 1991), long-term couples develop collaborative memory systems in which one partner often specializes in remembering relationship-related events while the other relies on them. Research suggests that in heterosexual couples, women are more likely to manage these memory domains, and men often depend on their partners as external memory aids (Niedźwieńska and Zielińska, 2021). Although our participants completed the questionnaires individually, their responses may still reflect a shared marital memory system shaped by ongoing interactions with their spouse. As such, individual reporting may mask gender differences by reflecting habits of mutual memory collaboration that have developed within the marital context. In summary, while gender differences in MR have been documented in some settings, their absence in our study likely reflects the influence of methodological design, shared memory dynamics embedded in marital life, and relationally driven motivational convergence.

These results concerning gender contrast with several studies that emphasize women’s stronger engagement with emotionally rich, relational, and well-rehearsed memories (e.g., Alea and Vick, 2010; McCoy et al., 2017). Such findings are often explained by socialization patterns, based on which women are more frequently engaged in emotion-focused storytelling, particularly with their mothers from early childhood (Fivush and Zaman, 2014; Reese et al., 1993), which contributes to their superior narrative and emotional memory skills in adulthood. Moreover, women tend to be more sensitive to relational cues and more affected by their partner’s behaviors (Guilbault and Philippe, 2017), which may intensify the role of reminiscence in shaping their marital satisfaction. However, the absence of gender-based differences in the present study aligns with findings from Philippe et al. (2013) and Lenger and Gordon (2019), which also reported no moderating role of gender in reminiscence–marital outcome relationships. One important factor that may account for this divergence is the use of structured self-report instruments in the current study. Unlike open-ended or narrative formats that allow for elaboration and emotional richness (which often amplify gender differences), standardized rating scales and closed-ended questions tend to reduce these differences by guiding participants toward similar types of responses (Grysman and Hudson, 2013). In this context, men may be more comfortable and capable of recalling and rating memories in ways comparable to women, resulting in more homogeneous gender responses.

In contrast, the significant age-related differences in NRS and IPSRS are consistent with previous studies suggesting that younger and midlife adults are more actively engaged in elaborative and emotionally intense reminiscence (Alea and Vick, 2010). This pattern may reflect the developmental relevance of such styles during phases of identity formation and relationship negotiation. The lack of age differences in frequency and ORS further suggests that not all forms of reminiscence decline with age, but that styles requiring emotional articulation and integrative meaning-making may be more susceptible to age-related changes. Studies reporting no age effects (e.g., Alea and Bluck, 2007) may have used broader or less differentiated memory constructs.

### Cultural framing of MRSs in Iran

While MRSs have been conceptually grounded in Western cultures, particularly in studies of older adults, their application in non-Western contexts like Iran requires culturally sensitive interpretation. Although general patterns may be consistent, the expression, function, and impact of MRSs are shaped by local socio-cultural norms.

In Iran’s collectivist and family-oriented society, marital memories are often interwoven with broader family and communal experiences. Key life events, such as weddings, childbirth, or family gatherings, commonly involve extended relatives and are framed as joint accomplishments or sources of pride. As a result, NRS and IPSRS in this context often highlight themes of loyalty, endurance, and shared responsibility within a larger familial system. Recalling these events can strengthen a couple’s sense of belonging to a supportive network, reinforcing their resilience and marital cohesion. Unlike individualistic societies where reminiscence may serve personal growth, in Iran, it may function more to reinforce family solidarity and collective meaning.

Cultural values also influence which styles are preferred or avoided. Social norms that emphasize emotional restraint, patience, and the avoidance of open conflict often discourage repetitive expression of distressing memories. Consequently, couples may consciously or unconsciously avoid OBRS, especially in shared settings. Instead, there is a cultural preference for recalling uplifting or redemptive memories that align with values such as forgiveness, unity, and perseverance. However, this can be double-edged. While such norms promote positive reminiscence and emotional regulation, they may also lead to suppression of unresolved pain or resentment. Clinical observations in Iran suggest that some partners may silently ruminate over past grievances without voicing them, leading to diminished psychological well-being despite outward harmony. As one Iranian participant in a qualitative study noted, *“Sometimes when we are alone, we recall good memories or the hardships we overcame together; this makes us feel more attached and helps us avoid dwelling on trivial issues that might harm our relationship”* (Golmohammadian and Salimi, 2023). This illustrates how NRS and IPSRS may be deliberately used to strengthen emotional bonds, while obsessive reflection on past hurts is culturally downplayed, even when its emotional toll remains unresolved.

Taken together, the Iranian cultural framework may shape not only which MRSs are culturally endorsed but also how these styles psychologically function. In this context, NRS and IPSRS may enhance psychological well-being not merely through cognitive reappraisal or emotional catharsis, as commonly posited in Western models, but through their alignment with culturally valued ideals such as endurance, unity, and moral continuity. Conversely, when culturally discouraged memories are internalized but not processed (as in silent rumination), the suppression of negative affect may erode well-being over time, despite surface-level marital harmony. Thus, cultural norms act as filters that both enable and constrain the pathways through which MRSs influences psychological outcomes.

### Implications of the study

This is the first study to utilize the MBRQ, opening a novel chapter for assessing reminiscence styles among married individuals. The development and application of this questionnaire provide researchers with a valuable tool to examine MR in diverse populations, enabling the generation of new insights across cultural contexts. By employing this questionnaire, researchers can accelerate their exploration of the role of reminiscence in marital relationships, identify previously unexplored associations, and propose new structural models. Furthermore, this tool can be particularly useful in intervention studies aimed at addressing MRSs. For example, it can measure shifts in MRSs following reminiscence therapy or other therapeutic interventions, allowing for a more detailed understanding of how these styles evolve and impact marital satisfaction. Overall, MBRQ facilitates the expansion of descriptive and correlational research in the field of inquiry, setting the stage for richer studies in marital dynamics.

In the therapeutic domain, this study underscores the importance of incorporating findings from descriptive and structural modeling studies into existing intervention protocols. It has already been found that memory building about positive events (as a component of savoring positive events) in couples lives results in increased relationship quality and dyadic adjustment (Costa-Ramalho et al., 2015). Moreover, Bazzini et al. (2007) showed that reminiscence of positive marital memories increases relationship satisfaction. By integrating insights from the assessment of MRSs, clinicians can refine therapeutic approaches to address specific MR patterns that influence positive marital outcomes. For instance, therapeutic protocols could include interventions that help couples redirect OBRS into more constructive, NRS that foster connection and understanding. Such enhancements to treatment protocols could lead to increased effectiveness in addressing marital challenges, ultimately improving relationship quality and psychological outcomes for couples. To further elaborate on how specific reminiscence styles may be targeted in intervention protocols, it is essential to consider their distinct psychological functions and relational dynamics. For instance, NRS can be deliberately evoked in couple-based therapy through structured activities that prompt partners to recall and share meaningful positive memories, such as the story of how they met, shared milestones, or moments of mutual joy. These interventions can foster emotional closeness, shared identity, and mutual appreciation. In contrast, OBRS, characterized by repetitive, negative, and rigid recall of distressing events, may require focused techniques such as emotional regulation training, cognitive reframing, and guided meaning-making exercises to reduce rumination and redirect attention toward more adaptive forms of reflection. Additionally, IPSRS, which involve revisiting past challenges to extract insight, meaning, or collaborative strategies, can be actively encouraged in therapy sessions. Therapists might guide couples to jointly reflect on how they overcame past difficulties and what personal or relational growth emerged from those experiences. This process can help build resilience, reinforce a shared narrative of coping, and increase satisfaction and cohesion within the relationship. By incorporating these differentiated interventions based on MRSs, clinicians can more effectively address relationship challenges and promote marital quality.

At the societal level, promoting descriptive and intervention studies on MRSs can significantly enhance public awareness and understanding of this concept. Educating couples about the role of MRSs in fostering marital intimacy could encourage them to adopt healthier patterns of reflection in their daily lives. By viewing MR as a practical tool for improving marital quality, couples may be empowered to actively incorporate positive reminiscence practices into their relationships. This increased familiarity with the benefits of MR could lead to broader societal improvements in relationship quality and satisfaction, contributing to stronger and more resilient families. In summary, this study highlights the potential for MRSs to enrich research methodologies, inform therapeutic strategies, and improve marital outcomes on a societal level. It offers a foundational framework for further exploration, while its practical applications have the potential to significantly impact couples’ lives.

### Limitations and suggestions for future research

This study has several limitations that should be addressed in future research. First, the reliance on self-report measures introduces the possibility of response bias, as participants may provide subjective or socially desirable responses. Incorporating qualitative methods, such as interviews or mixed-method approaches, in future studies could provide deeper insights and reduce potential biases. Second, the study was conducted in a single country within a specific cultural context, which limits the generalizability of the findings to other populations and cultural settings. To enhance cross-cultural understanding, future research should explore MRSs in diverse cultural contexts. Third, the questionnaire used to assess MRSs is relatively new, and its factor structure may evolve as it undergoes further validation. Normative studies across different populations are recommended to refine the scale through additional exploratory and confirmatory factor analyses, making it a more robust and widely applicable tool for future research. Fourth, one key limitation of the study was the use of convenience sampling, which may have limited the diversity of the sample in terms of education level, socioeconomic status, digital access, and general willingness to participate in psychological research. Future studies are recommended to employ more rigorous and representative sampling strategies, such as random sampling, to ensure broader inclusion. Fifth, the exclusive use of an online survey format may have introduced self-selection bias and response distortions, such as reduced attentiveness or socially desirable responding. These factors may have affected the representativeness and reliability of the data. Combining online data collection with alternative methods (e.g., in-person interviews or paper-based surveys) could help minimize bias and improve data quality.

## Conclusion

This study addressed five research objectives outlined in the introduction. First, the significant associations found between MRSs and marital quality support the hypothesis that how couples engage in reminiscing is closely tied to the perceived quality of their relationship. Second, the findings further revealed that MRSs are also related to individual psychological well-being, highlighting that reminiscing within a marital context can affect personal adjustment. Third, through structural equation modeling, we demonstrated that marital quality mediates the relationship between MRSs and PWB, indicating that reminiscence exerts its role in predicting well-being partly through its association with relational functioning. Fourth, no significant gender differences were observed in the use of MRSs, a finding that contrasts with some prior literature and was discussed in light of the characteristics of the current measurement tool. Fifth, the analysis revealed significant age-related differences in MRSs, particularly for NRS and IPSRS, suggesting that reminiscence styles may evolve with age and experience.

Therefore, this study offers several novel contributions to the literature. To our knowledge, it is the first to examine MRSs using a newly developed and validated instrument (MRQ), which moves beyond reminiscence frequency-based measures used in earlier research. Prior studies had mainly focused on the bivariate relationship between the frequency of marital reminiscing and marital outcomes. In contrast, the current study demonstrated that specific MRSs are associated with distinct relational and psychological outcomes, advancing our understanding from a simple frequency-based view to a more nuanced, qualitative perspective. Furthermore, this study was the first to examine the roles of gender and age in the application of MRSs, adding valuable insight into how such styles vary across individual characteristics. Beyond theoretical contributions, the findings were translated into practical guidelines, offering implications for incorporating MRSs into marital intervention protocols. Cultural considerations were also discussed to highlight how Iranian sociocultural norms might shape the function and impact of reminiscing within marriages. These integrated contributions collectively position this study as an important step forward in both theory and practice concerning MR processes.

## Data Availability

The datasets presented in this study can be found in online repositories. The names of the repository/repositories and accession number(s) can be found at: 10.17605/OSF.IO/ZN6SR, https://osf.io/zn6sr/.
